# Editorial

**Published:** 1902-04-15

**Authors:** 


### EDITORIAL.

At the January meeting of the New York City Odontological Society, Dr. A. W. Harlan was presented with a beautiful "loving cup," as an evidence of the esteem in which this well-known editor, teacher and practitioner from the great western metropolis, is held in the East. Dr. Harlan announced that he had attended twenty-one meetings of this society. Putting this incident and statement together, we are bound to conclude that there must be a reciprocal attachment, when a busy practitioner is willing to sacrifice so much valuable time and money as are involved in making a trip of a thousand miles to attend a dental society meeting once each year for twenty-one years. It is a record to be proud of, and the society honored itself in taking notice of it in an appropriate manner. Another reflection comes to us out of this incident which is not quite so pleasing to contemplate, but perhaps may be quite as profitable for some of us to think about seriously: We know some very good and honorable practitioners who are so engrossed in their own particular affairs that they are not willing to make a sacrifice of a few evening hours once each month to attend a dental society meeting in their own cities, and can scarcely be prevailed upon to attend their own State society, and never any of the national meetings. Executive officers and program committees have to resort to all sorts of schemes to coax out these diffident or unappreciative, stay-at-home members of the profession, but with scant success, for we find at all society gatherings the customary attendants with only a few new faces out of which perhaps one habitual or occasional recruit is secured to take the place of those who inevitably must drop out. If all members of the profession were endowed with a measure only, of Dr. Harlan's spirit, what grand gatherings of professional men we should have, and what wonderful progress our profession would make! If Dr. Harlan will open his heart and tell us what he finds in these gatherings that is so attractive, perhaps he may be the means of making society work attractive to others who do not now appreciate its value. Tell us why you do it, brother Harlan, and perhaps, if you don't make us ashamed of our excuses, we will give our reasons why we do not find great pleasure or profit in associated work.

THE *Dental Cosmos* has reprinted in pamphlet form the proceedings of the International Dental Federation meetings held in Paris, London and Cambridge. The addresses of Professors Foster and Griffiths, extracts of which we have published in the REGISTER, are well worth anyone's perusal, We presume copies may be obtained from the *Dental Cosmos* publishers.

